# Intra-coronary Imaging for the Evaluation of Plaque Modifications Induced by Drug Therapies for Secondary Prevention

**DOI:** 10.1007/s11883-020-00890-4

**Published:** 2020-10-06

**Authors:** Ismail Dogu Kilic, Enrico Fabris, Elvin Kedhi, Liviu-Nicolae Ghilencea, Gianluca Caiazzo, Sara Abou Sherif, Carlo Di Mario

**Affiliations:** 1 Department of Cardiology, Pamukkale University Hospitals, Denizli, Turkey; 2 Cardiovascular Department, University of Trieste, Trieste, Italy; 3 Department of Cardiology, Isala Heart Center, Zwolle, the Netherlands; 4 University of Medicine and Pharmacy Carol Davila, Bucharest, Romania; 5 P.O. San Giuseppe Moscati, Aversa, Italy; 6 Brighton and Sussex Medical School, Brighton, UK; 7 Cardio-toraco-vascular Department, Careggi University Hospital, Florence, Italy

**Keywords:** Coronary artery disease, Coronary atherosclerosis, Vulnerable plaque, Intra-coronary imaging, Secondary prevention

## Abstract

**Purpose of Review:**

Patients diagnosed with coronary artery disease are at a high risk of subsequent cardiovascular events; therefore, secondary prevention in the form of therapeutic lifestyle changes, and drug therapies is vital. This article aims to review potential application of intra-coronary imaging for the evaluation of plaque modifications, induced by medications for secondary prevention for CAD.

**Recent Findings:**

Intra-coronary imaging provides detailed information on the atherosclerotic plaque which is the primary pathological substrate for the recurrent ischemic cardiovascular events. These modalities can detect features associated with high risk and allow serial in vivo imaging of lesions. Therefore, intravascular imaging tools have been used in landmark studies and played a role in improving our understanding of the disease processes.

**Summary:**

Changes in size and plaque composition over time can be evaluated by these tools and may help understanding the impact of a treatment. Moreover, surrogate imaging end points can be used when testing new drugs for secondary prevention.

## Introduction

Patients diagnosed with coronary artery disease (CAD) are at a high risk of subsequent cardiovascular events. Modifications of risk factors can significantly reduce these recurrent events and premature death amongst these patients. For that reason, secondary prevention in the form of therapeutic lifestyle changes, and drug therapies is vital. The evaluation of treatment effect and risk stratification during follow-up is important because this may enable more intensive treatment, motivation and strict follow-up to reduce clinical outcomes. In addition to clinical outcomes, a number of tools are used to evaluate the impact of preventive strategies including biomarkers or imaging modalities. With regard to imaging of coronary atherosclerosis, intravascular modalities are able to provide high-resolution images that neither angiography nor other non-invasive modalities could. These techniques have been used in landmark studies and played a role in improving the understanding of the disease processes and the prevention strategies. Therefore, this article aims to review potential application of intra-coronary imaging (ICI) for the evaluation of plaque modifications induced by medications for secondary prevention for CAD.

## Why Is Intra-coronary Imaging Important?

### ICI for Better Definition of Atherosclerotic Coronary Disease

Atherosclerotic plaque is the primary pathological substrate leading to ischemic cardiovascular events, and interventions that have favourable effects on the atherosclerotic plaque are expected to improve clinical outcomes [1]. Therefore, atherosclerosis imaging can be utilized as an indicator of the ongoing pathologic process and response to therapeutic interventions. Coronary angiography has been the gold standard for detecting and guiding the treatment of atherosclerotic coronary artery disease. However, despite objective and reproducible measurements can be obtained by quantitative coronary angiography (QCA), it remains limited to merely reflecting the degree of lumen intrusion of atherosclerotic lesion in the contrast filled lumen. Especially in the early stages of the disease, plaques can show an outward growth, known as positive remodelling, without luminal compromise, not detectable by conventional angiography. Furthermore, the characteristics and composition of the plaque cannot be adequately studied by angiography, which only shows large calcifications and ulcerations. Finally various factors such as diffuse disease, vessel foreshortening, angulation, overlap, calcification, eccentricity and contrast streaming make angiographic assessment challenging [2]. Therefore, ICI modalities have been developed to overcome these limitations of conventional angiography. Moreover, ICI provides detailed evaluation of the plaques to discriminate between high- and low-risk lesions and/or patients. From pathological and clinical studies, we know that plaques at high risk for rupture share certain characteristics [3]. Various ICI modalities can detect these features associated with high risk and allow serial in vivo imaging of lesions, contributing to our understanding of the natural history of plaques and the effect of treatment. In fact, detecting high-risk features potentially helps in identifying patients in need of intensive anti-atherosclerotic regimens. The opposite is also true for the lower risk groups. If we can truly classify these patients, intensity of the treatments can be softened and decrease drug-related side effects, and repeated follow-up visits can be safely avoided allowing us to concentrate on truly high-risk individuals. Consequently, if we manage to focus our efforts in the group at higher risk, we may increase their compliance with regular follow-up visits and predict new acute events with frequent provocative tests [4]. Finally, atherosclerosis is a systemic disease; thus, focal therapies are by no means the main therapy. However, in the presence of an effective focal treatment strategy, to help reduce the number of patients with high-risk plaques, ICI may contribute by helping to identify a certain subset of patients who may benefit from these therapies, alongside systemic treatments [4].

### ICI for Evaluation of Treatment Plaque Modification

ICI provides a close look to the atherosclerotic plaque, which cannot be substituted by any non-invasive imaging. Consequently, changes in size and composition over time can be evaluated in matched arterial segments. These changes not only help understanding the impact of a treatment but can also be used as a surrogate clinical end point, which is important in testing new drugs [5]. Secondary prevention by means of vigorous risk modification and consistent use of antiplatelet and lipid-lowering agents according to guidelines significantly reduces event rates also in the placebo arm of trials testing new drugs, requiring studies on a larger numbers of patients over longer periods of time in order to reach a sufficient event rate to provide a sufficient statistical power and making the demonstration of an incremental effect of new agents more challenging [6]. On the other hand, surrogate imaging end points may allow a smaller group of patients to be studied for a shorter period of time and accelerate the drug development and analysis. This approach may indeed reduce the costs greatly and potentially prevent the risk of advancing inefficient therapies before further testing [7–9].

## Treatment and Plaque Modifications

### Statins

The clinical benefits of statins have consistently been demonstrated in numerous large-scale studies. They are regarded as one of the key drugs in the primary and secondary prevention of CAD [10–12]. Early statin trials employed conventional angiography to assess their effect on the atherosclerotic plaque, in an attempt to demonstrate that aggressive lipid-lowering therapy could halt the progression and even promote regression of coronary atherosclerosis [13–15]. However, results of early studies of lipid lowering showed a phenomenon that was later called “the angiographic paradox”. The improvements in lumen measurements were unexpectedly small and inconsistent when compared with the large reductions in the clinical event rates [16]. The awareness of the phenomenon of positive remodelling and the importance of plaque characteristic and composition explains why angiography should be replaced by ICI to address directly the plaque changes.

Several small studies with ICI demonstrated favourable effects of statins. In one of the earliest studies utilizing IVUS, Takagi et al. showed that pravastatin reduced progression of coronary artery atherosclerotic plaque [17]. An angioscopic study also demonstrated that lipid-lowering therapy by statins dramatically decreased the yellow grade of coronary plaque [18]. Benefits of statins, particularly high-intensity statin therapy, have been shown in further larger studies with ICI. The REVERSAL study comparing pravastatin 40 mg vs atorvastatin 80 mg for 18 months revealed a significant increase in IVUS-determined atheroma volume in pravastatin arm whereas no significant change in intensive therapy arm [19••]. Subsequently, the ASTEROID study showed, for the first time, that very intensive cholesterol lowering with rosuvastatin can even regress the atheroma burden (0.79% reduction in atheroma volume detected by IVUS) [20••]. This effect of intensive statin therapy was further explored in the SATURN. Despite the lower level of LDL-C and the higher level of HDL-C achieved with maximal doses of rosuvastatin compared with atorvastatin, both treatment arms resulted in significant but comparable regression of coronary atherosclerosis during 104 weeks of therapy [21••]. Around about two-thirds of study patients demonstrated a regression of atherosclerosis and a reduction in the primary efficacy end point, percent atheroma volume in atorvastatin and rosuvastatin groups found to be 0.99% and 1.22%, respectively. Both this frequency and level of regression were exceptional, as compared with the results of prior IVUS studies [21••].

Nonetheless, the fact that plaque regression observed in these studies are only modest (measuring approximately in 1% level) raises the question of how statin therapy is associated with a significant decrease in cardiovascular events with such small reductions in plaque burden [1].

A possible explanation could be the potential favourable changes in plaque composition. The effect of statins on plaque composition has been firstly investigated using IB-IVUS system by Kawasaki et al. and showed significant reduction in the lipid component [22]. In the IBIS-4 study, patients with STEMI were treated with high-intensity rosuvastatin (40 mg/day) over a 13-month period. Despite significant atheroma regression, no change in VH-IVUS defined percent necrotic core was observed during serial examinations [23•]. Likewise, in the radiofrequency-IVUS subset of SATURN including 71 patients, a change in necrotic core volume did not accompany regression of coronary atheroma with maximally intensive statin therapy [24].

**Fig. 1:**
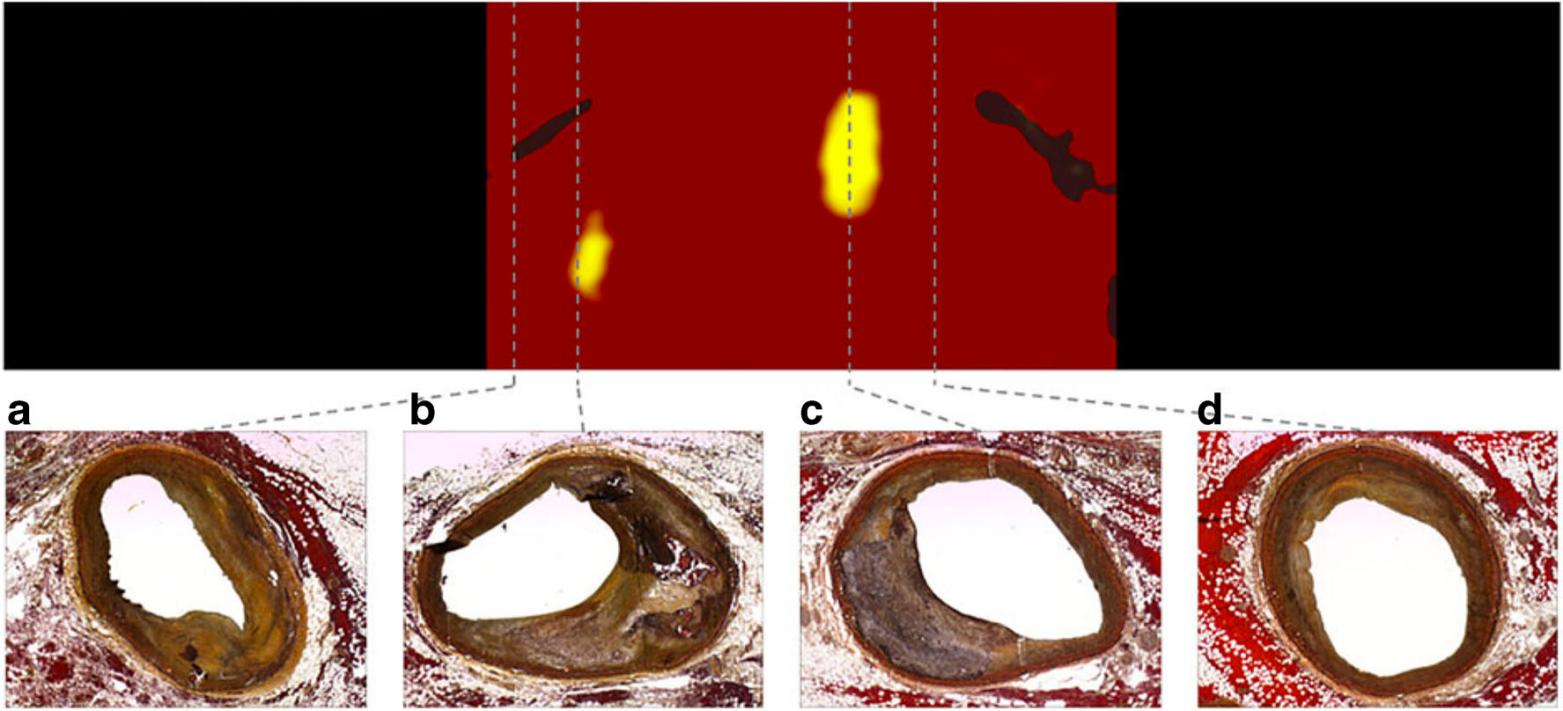
Example of correlation between NIRS chemogram and histologic findings. Vessel tissue lacking necrotic lipid core corresponds to ‘red’ in chemogram (**a** and **d**—fibrous PIT), whereas necrotic lipid core plaques correspond to ‘yellow’ (**b** and **c**—early stage fibroatheromas). Movat’s pentachrome stain used for histologic evaluation. (Adapted from: Kilic ID et al. Eur Heart J Cardiovasc Imaging. 2015;16(12):1299–306, by permission of Oxford University Press) [25]

Compositional changes with statins have also been investigated using near infrared spectroscopy (NIRS). NIRS can detect and quantify the presence of lipid core in the atherosclerotic plaque (Fig. 1), and NIRS-IVUS catheters may associate it with other features such as lumen size and plaque architecture [25]. Furthermore, NIRS imaging of non-obstructive coronary territories can aid in identifying patients and segments at higher risk for future events [26].

**Fig. 2:**
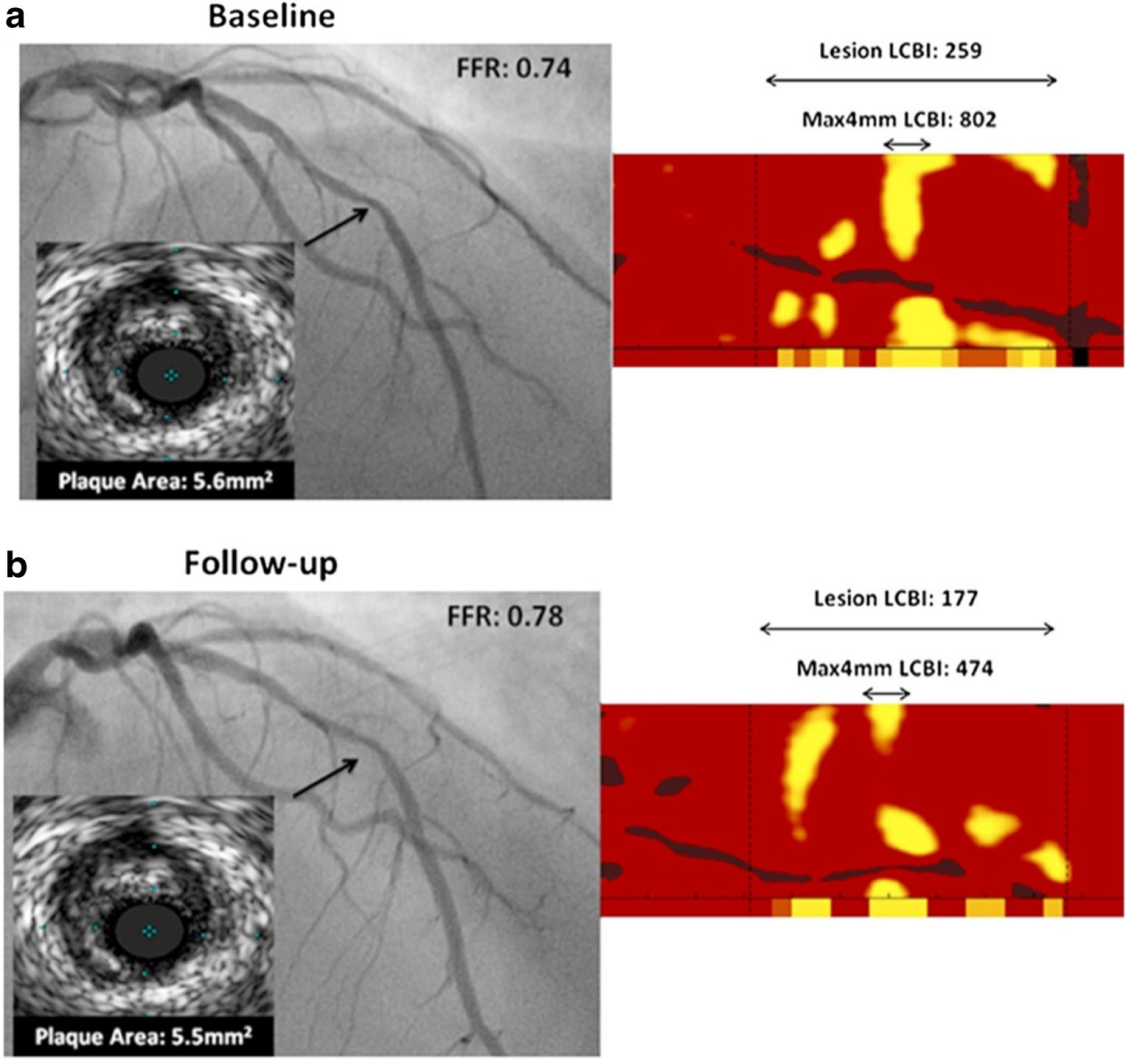
Top panel: On the left, the baseline angiographic image with FRR evaluation and the intravascular ultrasound cross-sectional images of the plaque are shown. On the right, the measurement of lesion lipid core burden index (LCBI) and LCBI at the 4-mm maximal segment (LCBI_4mm_ max) are shown. Bottom panel: On the left, the follow-up angiographic image with FRR re-evaluation and follow-up intravascular ultrasound cross-sectional image are shown. On the right, the reduction of lesion LCBI and LCBI_4mm_ max are shown. (Adapted from: Kini AS et al. J Am Coll Cardiol. 2013 Jul;62(1):21–9, with permission from Elsevier) [27]

The YELLOW trial randomized patients with multi-vessel CAD randomized to a treatment of either rosuvastatin 40 mg daily or the standard-of-care lipid-lowering therapy. Even after 6–8 weeks of a short-term intensive statin therapy, a significant reduction in the plaque lipid composition was demonstrated with maximum lipid core burden index in 4 mm (maxLCBI_4mm_) [27•] (see Fig. 2 for example illustrating IVUS and NIRS of a patient in the intensive group). In contrast, IBIS-3 study failed to demonstrate a significant reduction of necrotic core volume or LCBI under high-intensity rosuvastatin therapy for 1 year [28].

**Fig. 3:**
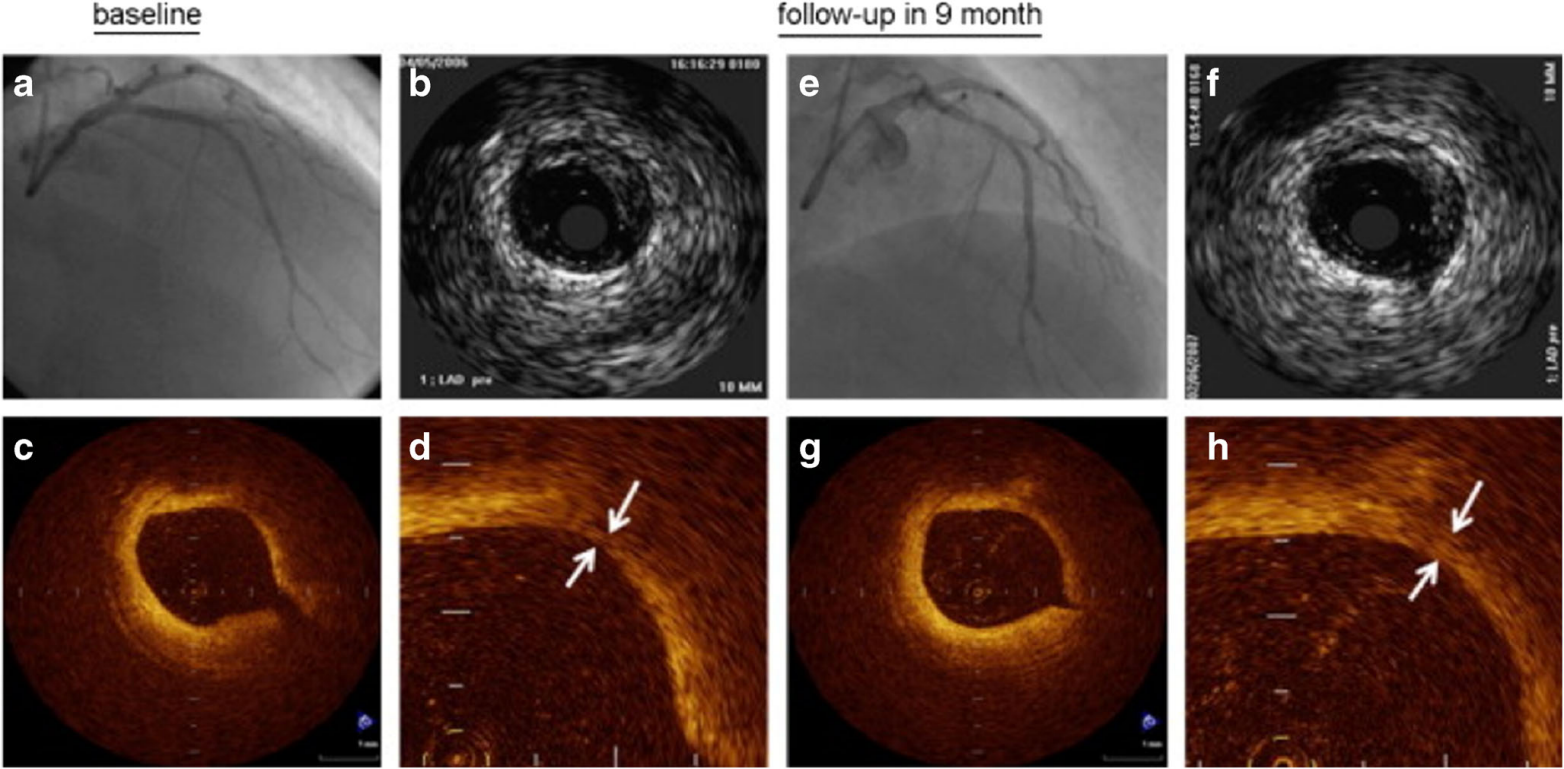
Coronary and intracoronary images of 47-year-old male patient in the statin treatment group. Four panels illustrate corresponding images with similar angle of coronary angiography (**a** and **e**) and corresponding cross-sectional images of IVUS (**b** and **f**), and OCT (**c**, **d**, **g**, **h**) at baseline and at 9-month follow-up. Fibrous cap thickness at the same cross-sectional image was increased from 110 (*white arrows* in D) to 320 m (*white arrows* in H) during 9-month follow-up period assessed by OCT. (Adapted from: Takarada S et al. Atherosclerosis. 2009 Feb; 202(2):491–7, with permission from Elsevier) [30]

Another important change in plaque composition is the calcification of the atherosclerotic plaque. In a recently published study, Puri et al. using IVUS data from a post hoc patient-level analysis of 8 prospective randomized trials on serial changes in coronary atheroma showed that statins promote calcification of the atheroma independently of their plaque-regressive effects [29]. Authors concluded that such results highlight the possible procalcific effects of statins, which may be related to possible plaque-stabilizing effects. Along with these findings, high-resolution OCT images introduced new possibilities into how statins act to stabilize plaques (Fig. 3).

In a study on 40 patients with previous myocardial infarction revealed that the degree of increase of fibrous cap thickness (FCT) was significantly greater in the statin treatment group than in the control group [30]. Recently, investigators showed that atorvastatin therapy at 20 mg/day provided a greater increase in FCT compared with 5 mg, which was associated with the decrease in serum atherogenic lipoproteins and inflammatory biomarkers [31]. In the YELLOW II study, which included 85 stable coronary artery patients, subjects were given 40 mg of rosuvastatin every day for 8–12 weeks. Baseline OCT minimal FCT was 100.9 ± 41.7 μm and significantly increased upon follow-up-FCT (108.6 ± 39.6 μm). The changes in FCT were also independently associated with the increase in cholesterol efflux capacity [32•].

### PCSK9 Inhibitors

Proprotein convertase subtilisin/kexin type 9 serine protease (PCSK9) plays a critical role in cholesterol metabolism. By binding to the LDL receptor and promoting their degradation and therefore reducing LDL uptake on the hepatocytes, PCSK9 increases LDL-C levels [33]. Moreover, PCSK9 might contribute to atherosclerosis by mechanisms independent of the LDL-C levels [34]. In the ATHEROREMO-IVUS study, serum PCSK9 levels were linearly associated with the fraction and amount of IVUS-VH necrotic core tissue [35]. Consequently, monoclonal antibodies against PCSK9 have emerged as a new and potent class of cholesterol lowering drugs. Recently, the GLAGOV trial evaluated the effects of the evolocumab on atherosclerosis with IVUS. Nine hundred sixty-eight patients with angiographic coronary disease were randomized to receive either evolocumab or placebo along with the statins for 18 months. Lower levels of cholesterol in the evolocumab group (36.6 vs 93.0 mg/dL) was associated with reduction in percent atheroma volume for evolocumab (− 0.95% vs + 0.05% in the placebo group) [36••].

Currently ongoing PACMAN-AMI (NCT03067844) study will investigate the effect of the PCSK9 inhibitor alirocumab in a total of 220 acute MI patients undergoing PCI in the infarct-related artery and receiving guideline-recommended high-intensity statin therapy. A serial, multi-vessel imaging study will be performed to determine the change in plaque volume, lipid parameters by NIRS, and macrophages and FCT by OCT at week 52.

### Raising HDL

Several epidemiologic studies provided robust evidence that low levels of high-density lipoprotein cholesterol (HDL-C) are a significant predictor of CV risk [37, 38]. Greater increases in HDL-C are associated with a significantly reduced atheroma progression and lower events even in patients using statins [39]. A pilot study in patients with acute coronary syndromes investigated the effect of recombinant apolipoprotein (apo) A-1 Milano [40], which rapidly mobilized cholesterol and thereby reduced atherosclerotic plaque burden in experimental atherosclerosis models [41]. In this study, the recombinant ApoA-I Milano/phospholipid complex (ETC-216) administered intravenously for 5 doses at weekly intervals resulted significant regression of coronary atherosclerosis as measured by IVUS [40]. Further analysis from this study showed that regression of atherosclerotic plaque was characterized by a concomitant shrinkage of the EEM, which results in a lumen size that is virtually unchanged [42]. In other words, large changes in atheroma volume can occur with only minor luminal changes, which can be undetectable by angiography, supporting the importance of ICI.

Another attractive strategy for raising HDL levels is via inhibiting cholesteryl ester transfer protein (CETP). Torcetrapib was the first CETP inhibitor evaluated in phase III clinical trials; however, despite a substantial increase in HDL-C and decrease in LDL-C, there was no significant decrease in the progression of coronary atherosclerosis when compared with atorvastatin monotherapy in the ILLUSTRATE trial [43]. Moreover, torcetrapib was discontinued after a large clinical study was terminated prematurely due to increased risk of mortality and morbidity rates [44]. However, in a post hoc analysis of ILLUSTRATE trial, the extent of HDL-C increase was found to be inversely related to torcetrapib treatment as well as the degree of regression of atherosclerosis measured by IVUS [45]. Moreover, regression of atherosclerosis was observed in patients who achieved the highest levels of HDL-C with torcetrapib, supporting the notion that the achievement of very high levels of HDL-C via CETP inhibition has the potential to generate functional HDL particles that participate in reverse cholesterol transport [45].

In a recently published study, CER-001 infusions, an engineered lipoprotein particle mimicking pre-beta HDL and consisting of a combination of recombinant human apolipoprotein A-I and two phospholipids, also failed to reduce coronary atherosclerosis on IVUS and QCA when compared with placebo [46]. Finally, in the ApoA-I Synthesis Stimulation and Intravascular Ultrasound for Coronary Atheroma Regression Evaluation (ASSURE) trial, patients with coronary artery disease will be treated over a period of 26 weeks, and change in percentage of atheroma volume will be assessed by IVUS compared with baseline (NCT01067820).

### Lipid Apheresis

LDL-apheresis is an extracorporeal procedure to remove LDL-C and lipoprotein particles and provides a robust improvement in plasma lipoprotein profiles. In a small study in patients with familial hypercholesterolemia, lipid apheresis in addition to medical therapy resulted in a significant increase in minimal lumen diameter by coronary angiogram and decrease in plaque area by IVUS at 1 year compared with medical therapy alone, suggesting a possible regression of atherosclerosis by LDL-apheresis [47].

### Other Systemic Therapies

Impact of other currently available, such as ezetimibe [48, 49] or experimental non-statin modifying drugs, such as acyl–coenzyme A:cholesterol acyltransferase (ACAT), has also been investigated with IVUS. The enzyme esterifies cholesterol in a variety of tissues, and inhibition of the ACAT may prevent excess accumulation of cholesteryl esters in macrophages. However, 2 studies failed to show that ACAT inhibition favourably alters coronary atherosclerosis as assessed by IVUS [50, 51].

Modification of risk factors other than lipid profile also has an impact on the atheroma progression. In PERISCOPE trial, pioglitazone treatment resulted in a significantly lower rate of progression of coronary atherosclerosis compared with glimepiride in patients with type 2 diabetes (DM) and coronary artery disease [52]. Moreover, in patients with DM, once atherosclerosis is established, this is associated with an increased extent, complexity, and a more rapid progression than seen in non-DM patients. In DM patients, certain characteristics beyond ischemia, such as coronary atherosclerosis burden, progression and plaque composition, may need to be considered for a more refined risk stratification in these high-risk patients [53]. Recently, it is reported that DM patients with angiographically intermediate coronary lesions remain at risk for future MACE events, including those patients with FFR negative lesions [54]. Thus, combining functional assessment with imaging techniques may be required to guide our treatment strategy in these patients with high-risk, fast-progressing atherosclerosis. The COMBINE (OCT-FFR) (NCT02989740) is enrolling patients to examine whether the addition of OCT plaque morphological evaluation to FFR hemodynamic assessment of intermediate lesions in DM patients will better predict MACEs and possibly lead to new therapeutic strategies [55].

In the CAMELOT trial which assessed 2 different drug regimens vs placebo in patients with CAD patients, IVUS showed progression in the placebo group, a trend toward progression in the enalapril group, and no progression in the amlodipine group [56]. In the STRADIVARIUS trial, after 18 months of treatment, the weight loss drug rimonabant has failed to show an effect on disease progression for the primary end point percent atheroma volume but showed a favourable effect on total atheroma volume, the secondary end point [57].

The effects of a selective oral inhibitor of lipoprotein-associated phospholipase A_2_, darapladib, were compared with of placebo in patients with CAD. This enzyme is expressed abundantly in the necrotic core and may contribute plaque vulnerability [58]. After 12 months, there were no significant differences between groups in plaque deformability with IVUS palpography or plasma high-sensitivity C-reactive protein. In the placebo-treated group, however, necrotic core volume increased significantly, whereas darapladib was found to halt this increase, without a significant difference in total atheroma volume [58]. Nonetheless, further clinical trials failed to show a reduction in events in both stable [59] and acute CAD [60].

### Local Interventional Therapies

**Fig. 4:**
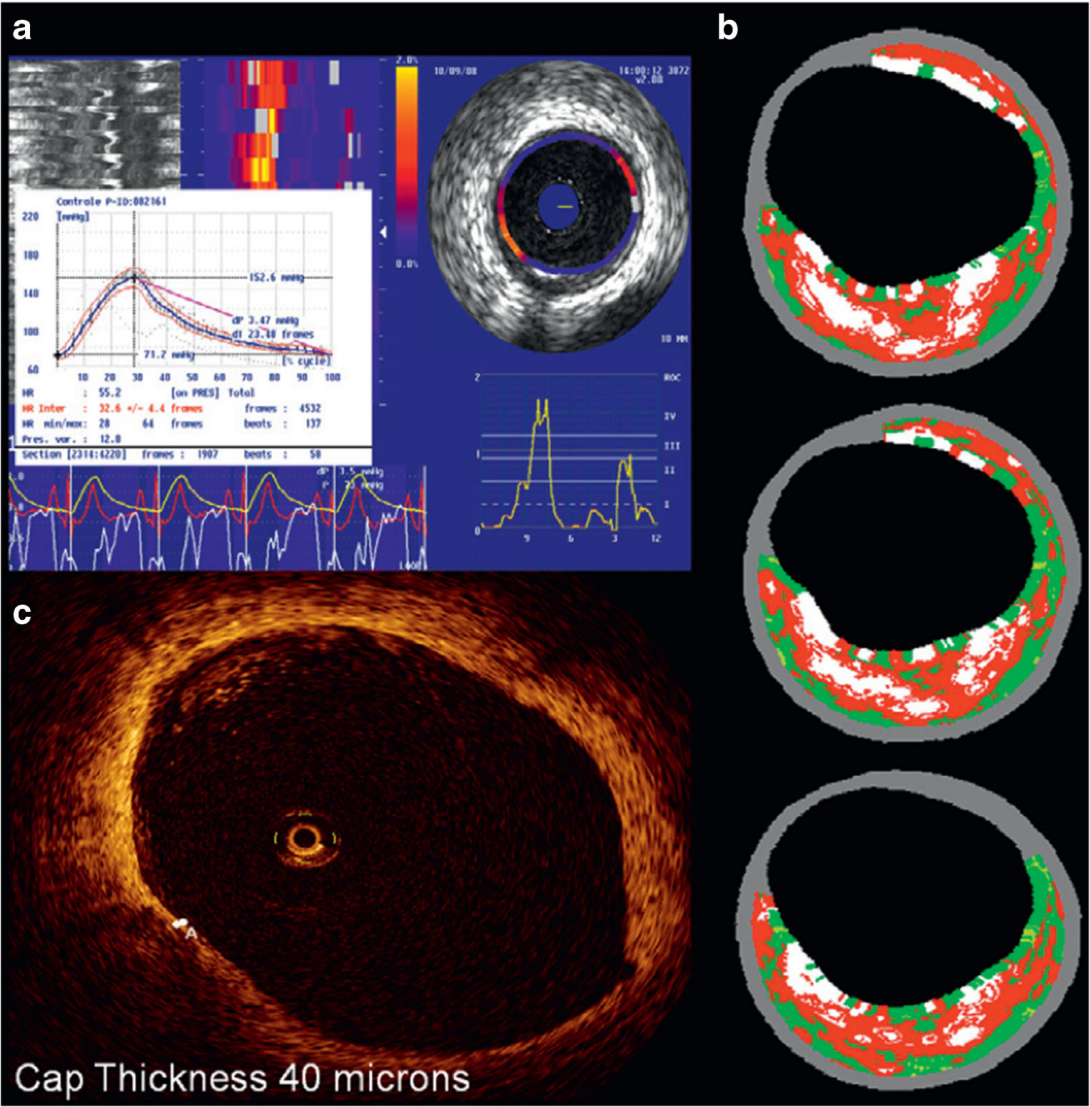
**a** In the upper left, palpogram showing stain value of 1.4% (Rotterdam Classification III-IV). **b** In the upper right, corresponding matched thin cap fibroatheroma on IVUS virtual histology analysis with plaque burden of 56% and necrotic core of 34% in three consecutive frames. **c** In the lower left corner, matched OCT frame showing cap thickness of 40 μm. (Adapted with permission of Europa Group from: Wykrzykowska JJ et al. EuroIntervention. 2012 Dec;8(8):945–54, permission conveyed through Copyright Clearance Center, Inc.) [61]

One of the potential treatment approaches under investigation for the high-risk plaques is the local treatment to sealing by the neo-intima formation which resembles a thick-cap and/or passivating the plaque by local immunomodulation. In this regard, ICI is not capable of detecting these “hot spots”, which carry a high risk, but additionally reveals any changes in vulnerable plaque characteristics with these treatments. A small pilot study tested the vShield self-expanding nitinol device (Prescient Medical, Inc., Doylestown, PA, USA), in which high-risk plaques were detected by VH-IVUS and OCT (see Fig. 4). The average baseline FCT, measured by OCT, was 48 ± 12 μm and increased to 201 ± 168 μm with neo-cap formation in the 6 months follow-up, which might contribute to stability of the plaque [61]. Similarly, using OCT, Brugaletta et al. showed the formation of a neointima layer after the implantation of a bioresorbable vascular scaffold that resembles a thick fibrous cap, which may contribute to stabilization of the plaque [62]. Although the concept is promising, it requires further investigation.

### Limitations

There are also limitations to the utilization of ICI for the studies. To start with, studies using ICI for surrogate end points needs to be interpreted with caution. This is because regardless of how promising a surrogate end point seems, the true clinical benefit may remain uncertain [63]. Also, there is always a possibility for unexpected off-target effects which may counterbalance the favourable effects. As mentioned previously, there are a number of examples where imaging markers showed benefit; however, subsequent clinical trials failed to show a clinical benefit or even showed harm.

Moreover, alterations with therapies do not always signify a clear-cut relation and/or causation. For instance, as previously mentioned, the reported effects of statins on plaque regression are modest, indicating that despite the statistical significance of this drug therapy, improvements in clinical outcomes may not be directly related to these minute changes in the vessel wall—so the mechanisms of the favourable effects of statins remained speculative.

Furthermore, despite the safety of these modalities are well documented [64, 65], they are not completely free of complications, and especially repeated examinations may be of concern with the use of ionizing radiation, contrast media and a need a vascular access.

Moreover, each individual modality provides information on different aspects of the lesion morphology and composition, complementing each other and otherwise lack perfection on their own. Additionally, a major limitation is the inability to assess specific biological processes with the currently available techniques. Molecular imaging techniques are also under development, with an aim to image-specific molecules and cells involved in the pathogenesis of disease using specialized, targeted imaging agents that bind to specific molecular or internalized within a cell [66–68]. In the future, these techniques may potentially allow the functional evaluation of the effect of therapeutic interventions on lesion activity.

There are also technical considerations on the use of ICI. First, acquiring good-quality images is not always possible; moreover, there are a number of artefacts which can preclude accurate measurements [7]. Secondly, matching the arterial segments can sometimes be challenging in different time points, although current co-registration systems may improve the correlations of different techniques. Another limitation is the need for predilatation to cross tight stenoses with the imaging catheter, which can significantly modify the lesion morphology and structure. This also hinders evaluation of totally occluded segments. Similarly, despite it is possible to evaluate the most of the epicardial coronary arteries, catheter-based imaging does not allow the visualization of the entire coronary bed [69].

## Conclusions

ICI provides detailed information on the atherosclerotic plaque, which is the primary pathological substrate for the recurrent ischemic cardiovascular events. These modalities have been showed to help investigating the effect of the medications for secondary prevention on the atherosclerotic plaque. Continuous advances in ICI may overcome current limitations and increase utilization of these modalities in the trials or even in risk stratification.
